# Induction of food-specific IgG by Gene Gun-delivered DNA vaccines

**DOI:** 10.3389/falgy.2022.969337

**Published:** 2022-09-19

**Authors:** Johanna M. Smeekens, Janelle R. Kesselring, Hannah Frizzell, Kenneth C. Bagley, Michael D. Kulis

**Affiliations:** ^1^ Department of Pediatrics, School of Medicine, University of North Carolina, Chapel Hill, NC, United States; ^2^ UNC Food Allergy Initiative, School of Medicine, University of North Carolina, Chapel Hill, NC, United States; ^3^ Orlance, Inc., Seattle, WA, United States

**Keywords:** food allergy, shrimp allergy, walnut allergy, CC027, C3H/HeJ, BALB/cJ, DNA vaccine, Gene Gun, IgG

## Abstract

**Background:**

Shellfish and tree nut allergies are among the most prevalent food allergies, now affecting 2%–3% and 1% of the US population, respectively. Currently, there are no approved therapies for shellfish or tree nut allergies, with strict avoidance being the standard of care. However, oral immunotherapy for peanut allergy and subcutaneous immunotherapy for environmental allergens are efficacious and lead to the production of allergen-specific IgG, which causes suppression of allergen effector cell degranulation. Since allergen-specific IgG is a desired response to alleviate IgE-mediated allergies, we tested transcutaneously-delivered DNA vaccines targeting shellfish and tree nut allergens for their ability to induce antigen-specific IgG, which would have therapeutic potential for food allergies.

**Methods:**

We assessed Gene Gun-delivered DNA vaccines targeting either crustacean shellfish or walnut/pecan allergens, with or without IL-12, in naïve mice. Three strains of mice, BALB/cJ, C3H/HeJ and CC027/GeniUnc, were evaluated for IgG production following vaccination. Vaccines were administered twice *via* Gene Gun, three weeks apart and then blood was collected three weeks following the final vaccination.

**Results:**

Vaccination with shellfish allergen DNA led to increased shrimp-specific IgG in all three strains, with the highest production in C3H/HeJ from the vaccine alone, whereas the vaccine with IL-12 led to the highest IgG production in BALB/cJ and CC027/GeniUnc mice. Similar IgG production was also induced against lobster and crab allergens. For walnut/pecan vaccines, BALB/cJ and C3H/HeJ mice produced significantly higher walnut- and pecan-specific IgG with the vaccine alone compared to the vaccine with IL-12, while the CC027 mice made significantly higher IgG with the addition of IL-12. Notably, intramuscular administration of the vaccines did not lead to increased antigen-specific IgG production, indicating that Gene Gun administration is a superior delivery modality.

**Conclusions:**

Overall, these data demonstrate the utility of DNA vaccines against two lifelong food allergies, shellfish and tree nuts, suggesting their potential as a food allergy therapy in the future.

## Introduction

Food allergies now affect 10% of the US population, greatly impacting the quality of life of patients and their caregivers (1–3). Annual costs to the US healthcare system are estimated at $25 billion per year (4). While some food allergies naturally resolve in the first few years of life, shellfish and tree nut allergies are often lifelong (5, 6). Approximately 2%–3% of the US population is allergic to shellfish, with the most prevalent allergies being to crustaceans, such as shrimp, lobster, and crab (1, 7). Importantly, there is a high degree of homology among the allergens across crustaceans. For example, tropomyosin from shrimp is 93% homologous to that of lobster (8). Approximately 1% of the population is allergic to tree nuts, with seed storage proteins being the major allergens (9, 10). Walnut and pecan allergens are highly homologous with one study demonstrating that walnut- and pecan-specific IgE having a correlation of 0.96 (11). Targeting allergens that are highly homologous could have broad applicability for the treatment of multiple food allergies (12–14).

Despite the increasing prevalence of food allergies, the mainstay of therapy is limited to strict dietary avoidance of allergens and access to epinephrine in case of an accidental exposure causing a reaction. In 2020, the FDA approved the first ever desensitization therapy for peanut allergy after a successful Phase 3 trial of peanut oral immunotherapy (OIT) (15). While approval of peanut OIT is a breakthrough for peanut allergy, there are currently no therapies for shellfish, tree nuts, or any other food allergies. Additionally, OIT has limitations including gastrointestinal side effects, required daily dosing, and transient desensitization (16). Immunologically, OIT induces significant increases in allergen-specific IgG and IgG4, which has inhibitory effects on mast cells and basophils *in vitro* (17). Animal models of food allergy have been used to demonstrate the function of allergen-specific IgG in blocking effector cell degranulation through FcɣRIIb (18). Since IgG is therapeutic in the context of food allergy, we aimed to utilize DNA vaccines to produce allergen-specific IgG against crustacean and walnut/pecan allergens in naïve mice.

Historically, DNA vaccines have been tested by intramuscular (i.m.) delivery of naked plasmid DNA, which is effective in small animals (19), but not as immunogenic in humans. Enhanced delivery modalities have been developed to increase DNA uptake and expression by targeted tissue cells. For example, electroporation uses electrical pulses to enhance uptake and expression over 100-fold (20–23) and has demonstrated efficacy in several clinical studies (24–32). Gene Gun delivery, another enhanced DNA delivery technique, uses a pressurized helium or hydrogen gas to propel dried DNA-coated gold microbeads into the epidermis and upper dermis of the skin (33–39). There are important differences between electroporation and Gene Gun delivery, two of which could be important for DNA-based food allergy therapies. First, Gene Gun delivery efficiently transfects professional antigen presenting cells (APCs) that express class II MHC (40–42) whereas i.m. delivery with or without electroporation does not (43, 44). This could allow Gene Gun delivery to more effectively target allergen-specific CD4^+^ T cells and antibody producing B cells. Second, whereas i.m. delivery with or without electroporation mainly transfects muscle cells and therefore primarily elicits systemic immune responses, Gene Gun delivery transfects epidermal cells making it a transcutaneous delivery technique that elicits mucosal immune responses in several mucosal compartments including the intestines and lungs (45–47). This could be a therapeutic advantage for Gene Gun delivery since food allergies trigger mucosal anti-allergen immune responses that may be targetable by a transcutaneous therapy but not by systemically delivered therapies like i.m. delivered DNA therapies.

DNA vaccines for peanut and Japanese Red Cedar allergies have been developed and tested in human trials (48). That DNA vaccine platform used lysosomal targeting of plasmid-expressed allergens by fusing the allergens to the lysosomal-associated membrane protein-1 (LAMP-1) that is a resident protein of the lysosome (49, 50). The attachment of the LAMP-1-targeting sequences to proteins in DNA plasmids directs the processing away from the class I MHC pathway towards the class II pathway (51, 52), leading to significantly enhanced immunogenicity of target antigens when delivered by Gene Gun. The LAMP-targeted DNA therapies for peanut and Japanese Red Cedar allergies showed promise in small animal models (49, 50), but were found to be suboptimal in humans (48).

DNA vaccines naturally evoke T_H_1 biased immune responses (53–55) making them ideal for allergen-specific therapeutic approaches. The T_H_1-biasing nature of DNA vaccines can also be enhanced by co-expressing T_H_1 cytokines with the vaccine antigens. IL-12 is a strong T_H_1-skewing cytokine (56, 57) and has been demonstrated to be a potent DNA vaccine adjuvant in small and large animal models (54, 58–64) and to be safe and effective in humans (25, 28, 65). For these reasons, we hypothesized that DNA-based allergy therapies could be dramatically enhanced by co-delivering them with IL-12 by Gene Gun. Here, we assessed Gene Gun-delivered DNA vaccines targeting either crustacean shellfish or walnut/pecan allergens in naïve BALB/cJ, C3H/HeJ, and CC027/GeniUnc mice for allergen-specific IgG induction.

## Materials and methods

### Mice

Four-week old female BALB/cJ and C3H/HeJ mice were purchased from Jackson Laboratories (Bar Harbor, ME). Four-to-six week old female CC027/GeniUnc mice were purchased from the UNC Systems Genetics Core Facility (Chapel Hill, NC). Mice were housed in a facility with a 12:12 light:dark cycle and kept on standard chow free of shellfish, tree nut, and peanut allergens. All studies were conducted under UNC IACUC protocol #21–044.

### Protein extracts

Lyophilized shrimp, lobster, and crab extracts were purchased from Greer Stallergenes (Lenoir, NC) and resuspended in PBS. Walnut and pecan extracts were prepared from flours (Holmquist Hazelnut Orchards, Lynden, WA) as previously described (66).

### DNA vaccines

**Figure 1:**
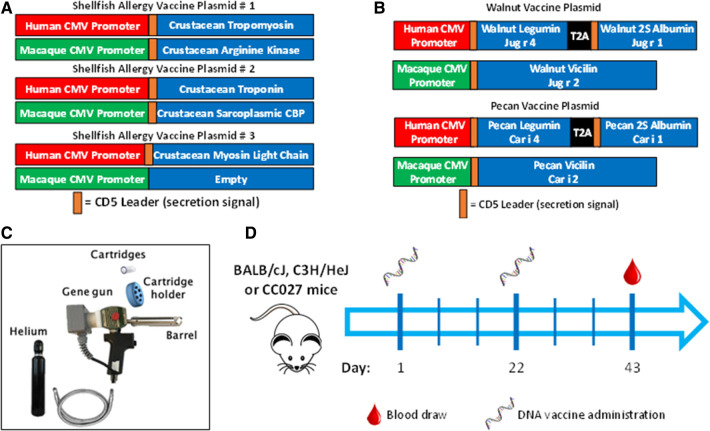
Vaccine formulation, delivery and schedule. DNA plasmid designs for (**A**) shellfish vaccine and (**B**) walnut/pecan vaccine. (**C**) Components of Gene Gun for transcutaneous vaccine administration. (**D**) Experimental scheme for vaccination.

The plasmid backbone used to construct the allergen-expressing plasmids is a dual promoter plasmid that has been used in several small and large animal DNA vaccine studies (25, 67) and has been evaluated in human clinical trials (25, 67). This plasmid has a human CMV promoter in the sense strand and a macaque CMV promoter in the opposite orientation in the opposing strand. The human and macaque CMV promoters express transgenes at similar levels. These different promoters were chosen to prevent the possibility of recombination events that could occur if two of the same promoters were incorporated into a single plasmid. The amino acid sequences of the walnut, pecan, and crustacean shellfish were obtained from the WHO/IUIS Allergen Nomenclature Database (allergen.org). The listed amino acid sequences for the walnut and pecan allergens were used, but for crustacean allergens, consensus sequences were derived using the consensus tool from the Influenza Research Database using input sequences from shrimp, prawn, lobster, crabs, and crayfish. Endogenous signal peptides were identified using SignalP and were replaced with human CD5 signal peptides to enhance secretion in human cells. The allergen amino acid sequences, including the CD5 signal peptides, were then human DNA codon-optimized by GeneWiz Inc. (South Plainfield, NJ). Those optimized DNA sequences were then synthesized and subcloned into the dual promoter plasmid backbone under the human or macaque CMV promoters as shown in Figures 1A,B by GeneWiz Inc. GeneWiz then verified the proper sequence of the allergens and their proper insertion into the plasmids by Sanger sequencing. Endotoxin-free plasmid maxipreps were made for vaccine use by Puresyn Inc. (Malvern, PA).

### Protein expression by mass spectrometry (MS)

For protein expression analysis, we used cell supernatants from allergen plasmid-transfected Expi293 cells. Briefly, the complete Expi293 Expression System was purchased from Thermo Fisher Scientific (Waltham, MA). The Expi293 cells were expanded in the supplied serum-free medium and then seeded into 6-well tissue culture plates. Individual wells of cells were then transfected with 2 *μ*g of each plasmid (walnut, pecan, or all three shellfish plasmids combined) using the supplied transfection reagent according to the Manufacturer's instructions. Forty-eight hours after transfection, the supernatants were removed from the transfected Expi293 cells, pooled within transfections and then centrifuged to remove cell debris. The clarified supernatants were then frozen at −20 °C for shipment to MS Bioworks (Ann Arbor, MI) on dry ice for analysis using their Protein-Works Protein Profiling platform.

### Vaccination with Gene Gun

Mice were vaccinated in the abdominal skin using a PowderJect XR DNA vaccine delivery system (referred to as the Gene Gun, PowderJect Vaccines, Inc., Madison, WI) as previously described (Figure 1C) (68). Briefly, mice were anesthetized with isoflurane, and abdominal fur was shaved with clippers prior to vaccination. Each DNA vaccination consisted of two tandem deliveries to non-overlapping areas of the abdominal epidermis. Each delivery consisted of 1 mg of 1–3-µm-diameter gold particles and 1–2 µg of total DNA. DNA vaccines were administered at a helium pressure of 400 lb/in^2^. Mice were administered vaccines on days 1 and 22, then bled *via* the submandibular vein on day 43 for antibody quantification (Figure 1D).

### Vaccination *via* intramuscular injection

Mice were vaccinated *via* intramuscular injection with electroporation on days 1, 15, and 29 for a total of three vaccinations and bled on day 43. Mice were injected in the hind quadricep muscle and the inoculations were immediately followed by *in vivo* electroporation using a BTX 2 needle array and a BTX ECM 830 Electroporation Generator (Holliston, MA) with the following parameters: six 100 V pulses with 50 ms duration and 200 ms between pulses.

### Sensitization to shrimp or walnut

Female BALB/cJ, C3H/HeJ, and CC027 mice were sensitized with shrimp or walnut extracts mixed with cholera toxin on days 1, 8, 15, and 22, followed by blood collection on day 36. Sensitizing doses were given by oral gavage with 2 mg food extract plus 10 µg cholera toxin.

### Immunoglobulin quantification

Shrimp-, lobster-, crab-, walnut-, and pecan-specific IgE, IgG, IgG1, IgG2a were quantified by ELISA, as previously reported (69). Briefly, plates were coated with 20 µg/ml food extracts (for samples) or 20 µg/ml HSA-DNP (for standard curves). After blocking with 2% BSA in PBS-0.05% Tween, serum samples were diluted 1:100 for IgE, 1:5,000 for IgG, 1:20,000 for IgG1 and 1:1,000 for IgG2a. Standard curves of mouse IgE anti-DNP, IgG1 anti-DNP or IgG2a anti-DNP (Accurate Chemicals, Westbury, NY) were generated ranging from 0.002–2 µg/ml. For IgE plates, the following detection antibodies were used in succession: 0.5 µg/ml sheep IgG anti-mouse IgE (The Binding Site, Birmingham, UK), 0.5 µg/ml biotinylated donkey IgG anti-sheep IgG (Accurate Chemicals), and 0.5 µg/ml NeutrAvidin-HRP (Pierce Biotechnology, Rockford, IL). For IgG, IgG1, and IgG2a plates, HRP goat anti-mouse IgG (Invitrogen, Waltham, MA), anti-mouse IgG1-HRP (Southern Biotech, Birmingham, AL), or anti-mouse IgG2a-HRP (Southern Biotech) were used, respectively. All plates were developed with TMB (SeraCare, Milford, MA), stopped with 1% HCl (SeraCare), and read on a plate spectrophotometer (BioTek, Winooski, VT) at 450 nm. Antigen-specific IgE, IgG1, and IgG2a concentrations were calculated based on the standard curve and dilution factor. Antigen-specific IgG is presented as O.D. values.

### Western blots

Shrimp, walnut and pecan extracts were separated on NuPage 4–12% Bis-Tris gels and transferred to nitrocellulose membranes before blocking with 2% BSA in PBS-0.05% Tween for 2 h at room temperature. Blots were incubated with pooled mouse serum (diluted 1:5,000 or 1:500 in 2% BSA PBS-0.05% Tween) overnight at 4°C with agitation. HRP goat anti-mouse IgG (Invitrogen) was diluted 1:5,000 in 2% BSA PBS-0.05% Tween and incubated with blots for 1 h at room temperature with agitation. Blots were developed with SuperSignal West Pico PLUS Chemiluminescent Substrate (Thermo Fisher Scientific), and imaged using an iBright imager (Invitrogen).

## Results

### Protein expression from DNA plasmids

Allergen protein expression from shellfish, walnut and pecan DNA plasmids were determined by mass spectrometry of secreted proteins from transfected Expi-293 cells. The major shellfish allergens, sarcoplasmic calcium-binding protein, troponin, tropomyosin, arginine kinase, and myosin light chain, encoded in the three DNA plasmids (Figure 1A) were all found to be highly expressed (Table 1). The walnut and pecan 11S legumin seed storage protein allergens Jug r 4 and Car i 4 were also highly expressed from their respective DNA plasmids (Figure 1B). By contrast, the 2S albumin seed storage protein allergens Jug r 1 and Car i 1 and the vicilin seed storage protein allergens Jug r 2 and Car i 2 were expressed at lower levels (Table 1).

**Table 1:** Expression of shellfish, walnut, and pecan allergens from DNA plasmids as determined by mass spectrometry.

Identified protein	MW in kDa	Relative abundance	% of highest expressing protein
**Expression of Shellfish Allergens (2,238 total proteins detected)**			
Heat shock 70 kDa protein	70	1349	100
Sarcoplasmic Calcium-Binding Protein	24	931	69
Troponin	22	610	45
Tropomyosin	35	498	37
Arginine Kinase	43	473	33
Myosin Light Chain	21	456	32
**Expression of Walnut Allergens (2,024 total proteins detected)**			
Heat shock 70 kDa protein	70	927	100
Jug r 4	58	729	79
Jug r 1	16	56	6
Jug r 2	70	10	1
**Expression of Pecan Allergens (4,685 total proteins detected)**			
Heat shock 70 kDa protein	70	1099	100
Car i 4	58	399	36
Car i 1	17	56	5
Car i 2	70	10	0.9

### Shrimp-specific immunoglobulin responses following vaccination

**Figure 2:**
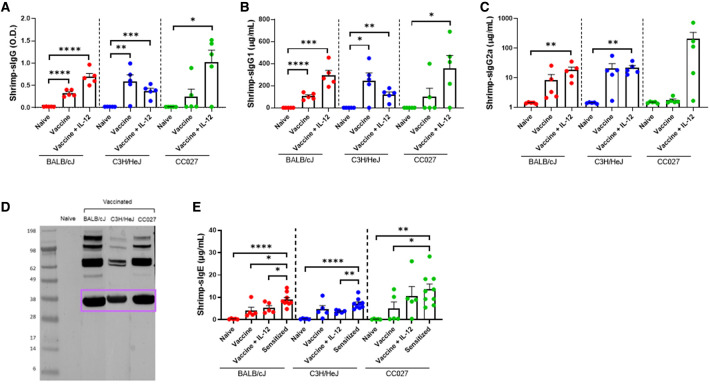
Shrimp-specific immunoglobulin responses following DNA vaccination with Gene Gun. Shrimp-specific (**A**) IgG, (**B**) IgG1, and (**C**) IgG2a in naïve and vaccinated BALB/cJ, C3H/HeJ and CC027 mice. (**D**) Western blot showing Gene Gun-vaccinated mice make shrimp-specific IgG against the major shellfish allergen tropomyosin (purple box). (**E**) Shrimp-specific IgE in naïve, vaccinated and sensitized BALB/cJ, C3H/HeJ, and CC027 mice. Statistical comparisons were made using unpaired *t* tests; *p < 0.05, **p < 0.01, ***p < 0.001, ****p < 0.0001.

Shellfish DNA vaccines were formulated onto gold microparticles with shellfish DNA plasmids alone or with mouse IL-12. Vaccines were administered transcutaneously *via* a PowderJect XR-1 Gene Gun (Figure 1C), which uses pressurized helium gas to propel dried DNA-coated gold microparticles into the epidermis. Naïve BALB/cJ, C3H/HeJ, or CC027 mice were vaccinated on days 1 and 22 and bled on day 43 (Figure 1D) to assess *in vivo* immunoglobulin production. Shrimp-specific IgG and IgG1 were significantly elevated in BALB/cJ and C3H/HeJ mice receiving either vaccine alone or vaccine with IL-12 compared to unvaccinated, naïve mice of the same strain (Figures 2A,B). CC027 mice produced increased quantities of shrimp-specific IgG and IgG1 following administration of vaccine with IL-12 as an adjuvant, but not when receiving the vaccine alone. Shrimp-specific IgG2a was produced in significantly higher quantities in BALB/cJ and C3H/HeJ mice that received the vaccine with IL-12 compared to naïve mice (Figure 2C). CC027 mice followed the same trend for shrimp-specific IgG2a. To determine which shrimp proteins the vaccine-induced IgG recognized, we used Western blotting. Importantly, the major shrimp allergen, tropomyosin (∼38 kD), was recognized by IgG induced in BALB/cJ, C3H/HeJ, and CC027, but not in unvaccinated naïve mouse sera (Figure 2D). In contrast, mice that received the vaccine alone or with IL-12 by intramuscular injection followed by electroporation did not make detectable levels of shrimp-specific IgG (Supplementary Figure S1).

To ensure that DNA-vaccinated mice produced limited quantities of shrimp-specific IgE, we quantified IgE from Gene Gun-vaccinated mice and compared these data to mice sensitized with shrimp plus cholera toxin. Vaccinated BALB/cJ mice made significantly less shrimp-specific IgE compared to sensitized mice (Figure 2E). On average, the vaccinated C3H/HeJ and CC027 mice produced less shrimp-specific IgE than their sensitized counterparts.

### IgG responses to additional crustaceans following shellfish vaccination

**Figure 3:**
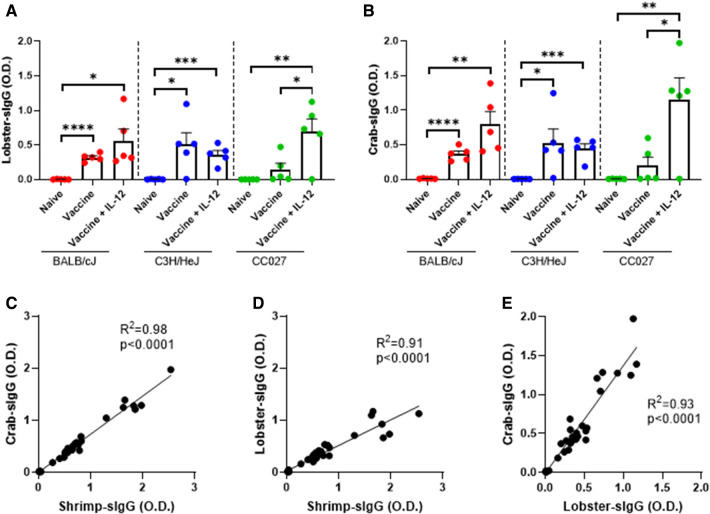
Lobster- and crab-specific IgG responses following DNA vaccination with Gene Gun. (**A**) Lobster-specific IgG and (**B**) Crab-specific IgG in naïve and vaccinated BALB/cJ, C3H/HeJ and CC027 mice. Correlations between (**C**) shrimp- and crab-specific IgG responses, (**D**) shrimp- and lobster-specific IgG responses, and (**E**) lobster- and crab-specific IgG responses in BALB/cJ, C3H/HeJ, and CC027 mice. Statistical comparisons were made using unpaired t tests; *p < 0.05, **p < 0.01, ***p < 0.001, ****p < 0.0001. Linear regression analyses were performed on the correlation plots.

Since there is a high degree of homology among crustacean allergens, we quantified IgG responses to lobster and crab from sera of mice vaccinated with the shellfish DNA vaccines (Figures 3A,B). Across all three strains of mice, the amounts of IgG produced were similar for shrimp, lobster, and crab within treatment groups (Supplementary Figures S2A–C). The correlation between crab- and shrimp-specific IgG was exceptionably high with an *R*^2^ of 0.98 (Figure 3C). Correlations between lobster- and shrimp-specific IgG and lobster- and crab-specific IgG also have *R*^2^ > 0.9, indicating the high degree of cross-reactivity between the IgG produced by the shellfish DNA vaccine (Figures 3D,E).

### Walnut-specific immunoglobulin responses following vaccination

**Figure 4:**
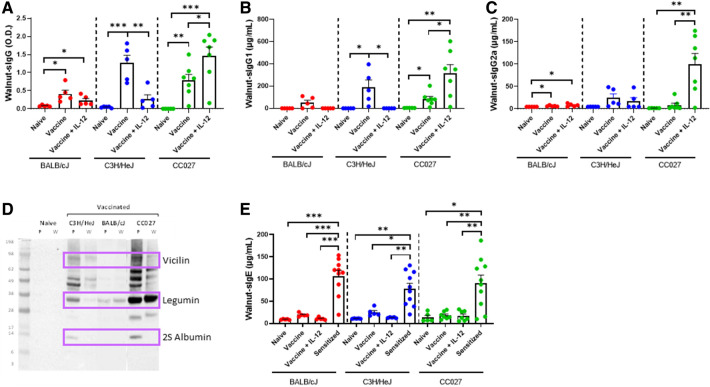
Walnut-specific immunoglobulin responses following DNA vaccination with Gene Gun. Walnut-specific (**A**) IgG, (**B**) IgG1, and (**C**) IgG2a in naïve and vaccinated BALB/cJ, C3H/HeJ and CC027 mice. (**D**) Western blot showing Gene Gun-vaccinated mice make walnut (**W**)- and pecan (**P**)-specific IgG against the major allergens (purple boxes): vicilin (Jug r 2 and Car i 2), legumin (Jug r 4 and Car i 4), and 2S albumin (Car i 1). (**E**) Walnut-specific IgE in naïve, vaccinated and sensitized BALB/cJ, C3H/HeJ, and CC027 mice. Statistical comparisons were made using unpaired *t* tests; *p < 0.05, **p < 0.01, ***p < 0.001.

To investigate the broad applicability of this DNA vaccination platform, we sought to apply our approach to walnut and pecan allergies, as an example for tree nut allergens. BALB/cJ, C3H/HeJ and CC027 mice were vaccinated *via* Gene Gun with walnut and pecan DNA plasmids following the same schedule as used for the shellfish vaccines (Figure 1D). Mice that were administered the walnut/pecan vaccine alone produced significantly higher levels of walnut-specific IgG compared to the respective naïve mice in all three strains (Figure 4A). BALB/cJ and CC027 mice that received the vaccine plus IL-12 also produced elevated levels of walnut-specific IgG compared to naïve mice, but this was not true for C3H/HeJ mice. Walnut-specific IgG1 production followed a similar trend, with C3H/HeJ and CC027 mice that received the vaccine alone having elevated levels compared to naïve mice (Figure 4B). CC027 mice that received the vaccine plus IL-12 also had significantly higher levels of walnut-specific IgG1 compared to naïve mice. Walnut-specific IgG2a production was most pronounced in the CC027 mice that received the vaccine plus IL-12, whereas the vaccine groups for the other strains made relatively low quantities of IgG2a (Figure 4C). IgG-binding proteins were identified by Western blot against walnut and pecan. We identified bands at ∼75, ∼33, and ∼12 kD corresponding to the vicilin (Jug r 2 and Car i 2), legumin (Jug r 4 and Car i 4), and 2S albumin (Car i 1) (Figure 4D), respectively.

Walnut-specific IgE was quantified in vaccinated mice and compared to mice that were sensitized with walnut plus cholera toxin. In all three strains, the sensitized mice produced significantly higher quantities of walnut-specific IgE compared to both vaccinated groups and the naïve groups (Figure 4E).

### Pecan-specific IgG responses following vaccination

**Figure 5:**
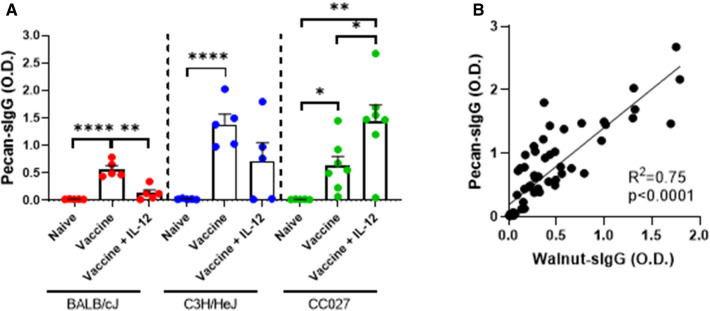
Pecan-specific IgG responses following DNA vaccination with Gene Gun. (**A**) Pecan-specific IgG quantities in naïve and vaccinated BALB/cJ, C3H/HeJ, and CC027 mice. (**B**) Correlation between walnut- and pecan-specific IgG responses. Statistical comparisons were made using unpaired *t* tests; *p < 0.05, **p < 0.01, ****p < 0.0001. Linear regression analysis was performed on the correlation plot.

Since the walnut/pecan vaccine contained both walnut and pecan DNA plasmids, we next investigated the quantity of pecan-specific IgG produced by vaccinated BALB/cJ, C3H/HeJ and CC027 mice. In all three strains, mice that received the vaccine alone had significantly higher pecan-specific IgG compared to naïve mice (Figure 5A). Including IL-12 in the formulation only elevated IgG production in the CC027 mice compared to the vaccine alone. Overall, pecan-specific IgG production was comparable to walnut-specific IgG across all three strains (Supplementary Figures S3A–C). Indeed, there was high correlation between walnut- and pecan-specific IgG (*R*^2 ^= 0.75) amongst all mice (Figure 5B).

## Discussion

Mechanistic studies from food allergen immunotherapy trials have demonstrated the importance of allergen-specific IgG in positive clinical outcomes (17, 70). IgG plays dual roles by either intercepting allergen before binding to cell surface bound-allergen-specific IgE or binding to inhibitory receptors, including FcɣRIIb, on the surface of effector cells. Studies have demonstrated that post-OIT and -SLIT plasma, containing high quantities of peanut-specific IgG, inhibits peanut IgE-mediated reactions *in vitro* (18, 71, 72). Therapeutically, IgG directed against defined allergen epitopes may abrogate, or greatly reduce, IgE-mediated reactions. This was recently demonstrated in a small clinical study that tested administration of two monoclonal IgG4 antibodies against distinct epitopes of the major cat allergen, Fel d 1 (73). A single injection of these antibodies reduced symptoms following nasal allergen challenge, demonstrating the utility of IgG directed against allergens as a therapeutic approach.

LAMP-targeted DNA therapies for peanut and Japanese Red Cedar allergies were suboptimal in humans, although the exact reasons are unknown. One potential shortcoming is that the LAMP-targeted DNA platform was based on simple naked DNA inoculation resembling the early naked DNA vaccines that also failed in human trials. Second, the therapy did not use any immunomodulators to tolerize the allergic immune responses or to change the nature of the responses from IgE-dominated T_H_2 responses to IgG-dominated T_H_1 responses. Third, i.m. delivery does not generate mucosal immune responses and likely does not have a profound impact on existing mucosal anti-allergen immune responses. The DNA vaccines tested in our work are not naked DNA, rather DNA plasmids coated on gold microparticles. We also utilized IL-12, a T_H_1 skewing adjuvant, and delivered the vaccines transcutaneously *via* Gene Gun, which allows for efficient transfection of skin APCs (36, 74–77). Overall, our approach has addressed each of these potential shortcomings of the LAMP-targeted therapy.

We utilized DNA vaccines to induce allergen-specific IgG production by targeting major allergens that have high homology across species. For shellfish, the DNA plasmids encoded consensus sequences of the crustacean allergens: tropomyosin, arginine kinase, troponin, sarcoplasmic calcium-binding protein, and myosin light chain. For the walnut/pecan vaccine, the DNA plasmids encoded specific allergens for walnut and pecan: Jug r 1, Jug r 2, Jug r 4, Car i 1, Car i 2, and Car i 4. There was high expression of Jug r 4 and Car i 4, but lower expression of Jug r 1, Car i 1, Jug r 2 and Car i 2 in Expi293 transfected cell supernatants. Expression of shellfish, walnut, and pecan antigens confirms that the selected DNA plasmids could serve as a potential vaccine, although increasing the production of Jug r 1, Car i 1, Jug r 2 and Car i 2 from the vaccine may be beneficial.

In mice, vaccines were transcutaneously administered by Gene Gun twice, three weeks apart, to prime and then boost the immune response. DNA plasmids were administered alone, or in combination with a mouse IL-12 plasmid to investigate the ability of a T_H_1-skewing adjuvant to enhance IgG responses. Interestingly, IL-12 did not have a universal effect, but appeared to further increase IgG responses in the CC027 strain, which are deficient in IL-12 production (78). Inclusion of IL-12 in the vaccine likely provided the necessary cytokine required to promote IgG production in CC027 mice, although there was variability in the IgG responses, possibly due to varying endogenous IL-12 production in each mouse. Overall, these vaccines administered *via* Gene Gun were successful at inducing allergen-specific IgG against the target foods, however, future studies may assess additional adjuvants and delivery regimens to enhance IgG production.

The data presented here are encouraging for the potential therapeutic use of DNA vaccines in food allergy. One major advantage of DNA vaccines is that any antigen with a known DNA sequence can be readily made into a vaccine. This is especially useful for allergens that have high sequence homology, since a vaccine directed against one highly conserved sequence can potentially be protective against allergens from multiple species. Using DNA vaccines applied to the skin may also lead to less severe side effects compared to therapies like OIT that are applied to mucosal surfaces like the gastrointestinal tract. Another advantage is that immunoglobulin responses are observed after only two vaccinations, compared to the daily dosing that is required of oral and sublingual immunotherapy. Potential limitations of DNA vaccines include the induction of IgE and side effects once the allergens are expressed *in vivo*; however, these are limitations with any allergen-specific immunotherapy. Overall, DNA vaccines are attractive potential therapeutics that warrant further investigation in food allergy.

In conclusion, DNA vaccines targeting shellfish, walnut, and pecan allergens induced antigen-specific IgG in three distinct genetic backgrounds of mice. These vaccines will next be investigated in mice sensitized to shellfish or tree nuts to test their potential therapeutic efficacy. Importantly, transcutaneous administration with Gene Gun was superior to i.m. administration with electroporation, which demonstrates the potential for this new route of administration of allergy therapies in future clinical trials. Successful DNA vaccines with strong safety and efficacy profiles would alter the treatment landscape for food allergy.

## Data Availability

The raw data supporting the conclusions of this article will be made available by the authors, without undue reservation.
