# Supplementary material for: Induction of food-specific IgG by Gene Gun-delivered DNA vaccines
Source: Front Allergy. 2022 Sep 19;3:969337. doi: 10.3389/falgy.2022.969337 (PMC9632862; doi:10.3389/falgy.2022.969337)
Supplement: Supplementary file 1 [file Datasheet1.docx]

Supplementary Material

# Supplementary Figures

**Supplementary Figure 1**. Shrimp-specific IgG1 in CC027 mice vaccinated with the shellfish DNA vaccine via intramuscular injection with electroporation.

**Supplementary Figure 2**. Crustacean-specific IgG responses following DNA vaccination with Gene Gun. Shrimp-, lobster- and crab-specific IgG quantities (S, L, C, respectively) in naïve and vaccinated (A) BALB/cJ, (B) C3H/HeJ, and (C) CC027 mice.

**Supplementary Figure 3**. Walnut- and pecan-specific IgG responses following DNA vaccination with Gene Gun. Walnut- and pecan-specific IgG quantities (W, P, respectively) in naïve and vaccinated (A) BALB/cJ, (B) C3H/HeJ, and (C) CC027 mice.
